# Correlation between severity of spinal stenosis and multifidus atrophy in degenerative lumbar spinal stenosis

**DOI:** 10.1186/s12891-021-04411-5

**Published:** 2021-06-12

**Authors:** Gen Xia, Xueru Li, Yanbing Shang, Bin Fu, Feng Jiang, Huan Liu, Yongdong Qiao

**Affiliations:** 1 Orthopedics, Medical Experiment Center, General Hospital of Ningxia Medical University, Yinchuan, China; 2 Department of Medicine, Aab Cardiovascular Research Institute, University of Rochester School of Medicine and Dentistry, Rochester, NY USA

**Keywords:** Degenerative lumbar spinal stenosis, Multifidus atrophy, Spinal stenosis TCSA/TFCSA ratio, Claudication distance

## Abstract

**Background:**

Degenerative lumbar spinal stenosis (DLSS) is a common degenerative condition in older adults. Muscle atrophy (MA) is a leading cause of muscle weakness and disability commonly reported in individuals with spinal stenosis. The purpose of this study was to investigate if the MA correlates with the grade of spinal stenosis in patients with DLSS.

**Methods:**

A retrospective analysis on 48 male and 184 female DLSS patients aged around 54.04 years (54.04 ± 8.93) were involved and divided into 6 groups according to claudication-distance-based grading of spinal stenosis, which confirmed by two independent orthopedic surgeons using T2- weighted images. Using 1.5T MRI scanner, the severity of MA is assessed based on its negative correlation with the ratio of total fat-free multifidus muscle cross-sectional area (TFCSA) to total multifidus muscle cross-sectional area (TCSA). Adobe Photoshop CS6 was used for qualitative image analysis and calculate the TFCSA/TCSA ratio to assess the severity of MA, compare the grade of MA with the spinal stenosis segment, stenosis grade and symptom side.

**Results:**

In DLSS group, The TFCSA/TCSA ratio are 74.33 ± 2.18 in L3/4 stenosis, 75.51 ± 2.79 in L4/5 stenosis, and 75.49 ± 2.69 in L5/S1 stenosis. there were significant decreases in the TFCSA/TCSA ratio of stenotic segments compared with non-stenotic segments of the spinal canal (*P <* 0.05) while no significant difference between the non-stenotic segments (*P > *0.05). TFCSA/TCSA ratios is significant differences in the TFCSA/TCSA ratios of the 6 DLSS groups (*F* = 67.832; *P < *0.05). From Group 1 to Group 6, the TFCSA/TCSA ratio of stenotic segments positively correlated with the absolute claudication distance (ACD). (*P* < 0.001, *r* = 0.852). Besides, the TFCSA/TCSA ratios are smaller in the symptomatic sides of the spine than the contralateral sides (*t* = 4.128, *P* = 0.001).

**Conclusions:**

The stenotic segments of the spinal canal are more atrophied than the non-stenotic segment in DLSS patients. It is shows that a strong positive correlation between the severity of multifidus atrophy and the severity of spinal stenosis.

## Introduction

Degenerative Lumbar Spinal Stenosis (DLSS) is an age-related condition in which the spinal canal narrows due to degenerative changes in the facet joint [1]. The degenerative narrowing of the spinal canal will induce compression of the vascular structures and ischemia of the spinal nerves, leading to low back pain (LBP), leg pain, neurogenic claudication, disability, and loss of independence [2–4]. DLSS is a chronic disease prevalent among aged adults [5]. A recent study shows that DLSS presented in up to 8.0 % of adults aged 50 years or older, and the incidence increases with advancing age [6], while another recent study involving sixty-seven individuals shows that DLSS is found on magnetic resonance imaging in more than 20 % of individuals aged more than 60 [7]. Typical symptoms of DLSS include pain in the groin, hips, and buttocks [1, 7]. The destructive impacts of DLSS on the ability to walk and move independently significantly diminishes the life quality of aged adults. Consequently, order patients desires for mobility and functionality have resulted in an increasing number of surgical intervention [8]. The increasing rate is even more dramatic over recent decades due to the increasing aged population, which emphasize the importance for clinicians to better understand the etiology and pathogenesis of DLSS [8–10].

Previous studies on the etiology and pathogenesis of DLSS have mostly focused on the degeneration of the three-joint complex with little attention to the role of muscles. However, recent studies have demonstrated that muscle atrophy is closely associated with DLSS [11]. Muscle atrophy (MA) is the loss of muscle mass and strength caused by immobility, aging, malnutrition, medications, injuries, or diseases [12]. It leads to muscle weakness, resulting in the inability to perform physical tasks [13]. Like DLSS, muscle atrophy is another pathological condition prevalent in aged adults highly associated with LBP [14, 15]. Multifidus atrophy has been found in > 80 % of patients with LBP, and its severity correlates directly with maintain of LBP symptoms [15, 16]. However, this linkage has not been examined yet. However, the correlation between multifidus atrophy (MA) and spinal stenosis remains poorly understood even though multiple studies have suggested the relationship between multifidus muscle and degenerative diseases of the lumbar spine [15].

Therefore, the purpose of this study is to evaluate whether there is a correlation between the severity of MA and the severity of spinal stenosis in DLSS patients to broaden our knowledge about the etiology and pathogenesis of DLSS.

## Materials and methods

### Study participants

Retrospectively, this study was conducted in General Hospital of Ningxia Medical University from January 2018 to April 2020. A total of 232 cases (48 males and 184 females) with a mean aged of 54.04 were involved as the DLSS group. These patients were diagnosed with DLSS based on 1.5 T magnetic resonance imaging (MRI) and medical history. In the DLSS group, 22 patients (15 females and 7 males) were diagnosed with L3/4 stenosis; 172 patients (120 females and 52 males) were diagnosed with L4/5 stenosis; 28 patients (16 females and 12 males) were diagnosed with L5/S1 stenosis. A control group of 60 non-DLSS patients (12 males and 48 females) with a mean age of 48.08 were also involved. The cases were selected following the ethics and approved protocol of the Committee of the General Hospital of Ningxia Medical University with written informed agreements obtained from patients. The summary of case information can be found in Table 1.

**Table 1:** Patient characteristics of the DLSS group and the control group

Variables	DLSS group	Control group	*P* value^*^
Age	54.04±8.93	48.08±8.93	>0.05
Sex, female	184/232 (79.3%)	48/60 (60%)	-
Sex, male	48/232 (20.7%)	12/60 (40%)	-
Medical history(mon)	6.60±2.57	-	-
L3/4 stenosis	22/232 (9.5%) (F,15; M,7)		
L4/5 stenosis	172/232 (74.1%) (F,120; M,52)		
L5/S1 stenosis	28/232 (12.1%) (F,16; M,12)		

^*^t test; Data is presented as mean ± standard deviation; Medical history, how long the patient has been diagnosed with DLSS; mon: month; F: female; M:male; *P* < 0.05 was considered to show a statistically significant difference

Inclusion criteria: (1) diagnosed as single-segment central canal stenosis or lateral recess stenosis based on clinical manifestations and imaging examination; (2) completed imaging data such as lumbar MRI; (3) multifidus muscle atrophy is the primary observation on MRI.

Exclusion criteria: (1) with nerve root canal stenosis, lumbar spinal stenosis, multi-segment lumbar spinal stenosis, or lumbar spinal stenosis secondary to lumbar spine instability; (2) with incomplete imaging data; (3) accompanied by other spinal related diseases, such as spinal tumors, spinal deformities; (4) with previous lumbar fractures or surgical history.

### Magnetic resonance imaging

Magnetic resonance imaging (MRI) of the lumbar spine was performed using a 1.5 T MRI scanner (MAGNETOM® Verio, a Tim + Dot System, Siemens, Erlangen, Germany) with participants in the supine position. Images were taken using the following parameters: sagittal T2-weighted images from T12 to the sacrum (TR/TE 2980/122.6, matrix size 208 × 320, time to recovery: 3,000–3,600 ms, time to echo: 87–114 ms, slice thickness: 4 mm) and axial T2-weighted images from T12 to S1 (TR/TE 2980/122.6, matrix size 208 × 320, time to recovery: 3,000–3,600 ms, time to echo: 87–114 ms, slice thickness: 4 mm).Two kinds of images were taken using the following parameters.

### Evaluation of multifidus atrophy

**Fig. 1:**
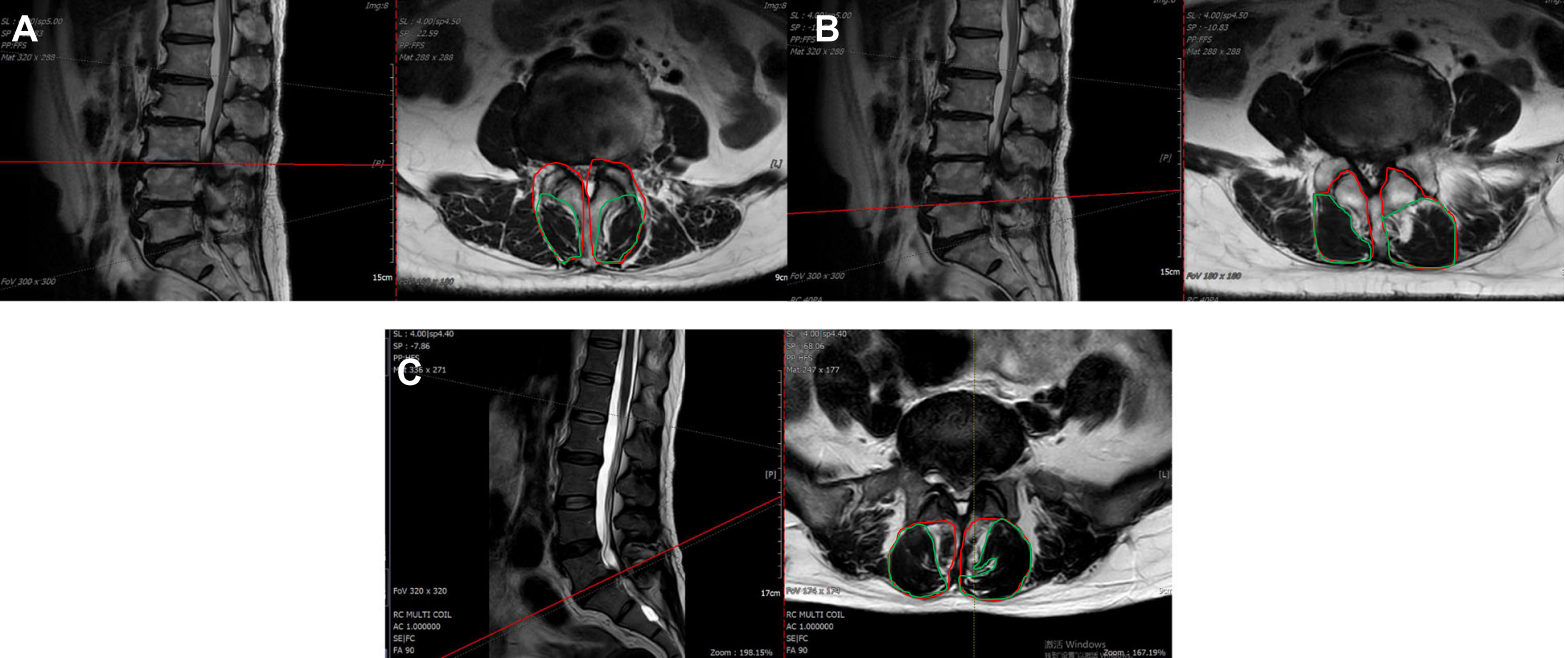
Measurements of TCSA and TFCSA at the L3/L4 (**A**), L4/L5 (**B**), and L5/S1(C) intervertebral discs. Red margins outline the multifidus muscle cross-sectional area (TCSA), while green margins outline the fat-free multifidus muscle cross-sectional area (TFSCA)

Total multifidus muscle cross-sectional area (TCSA) and total fat-free multifidus muscle cross-sectional area (TFCSA) were measured at the L3/L4, L4/L5, and L5/S1 intervertebral discs on both sides of the spine based on the semiquantitative grade system published by Patrick (Fig. 1). Image Proplus was used as the image processing software to outline and quantify TCSA and TFCSA. The severity of multifidus atrophy (MA) was assessed by calculating the ratio of TFCSA to TCSA (TFCSA/TCSA). Smaller TFCSA/TCSA indicates increased fat infiltration and more severe MA. We used Adobe Photoshop CS6 (Adobe Systems, San Jose California, USA) for qualitative image analysis. The ratio of TFCSA to TCSA (TFCSA/TCSA) was calculated to estimate the severity of MA.

### Grading of lumbar spinal stenosis

**Fig. 2:**
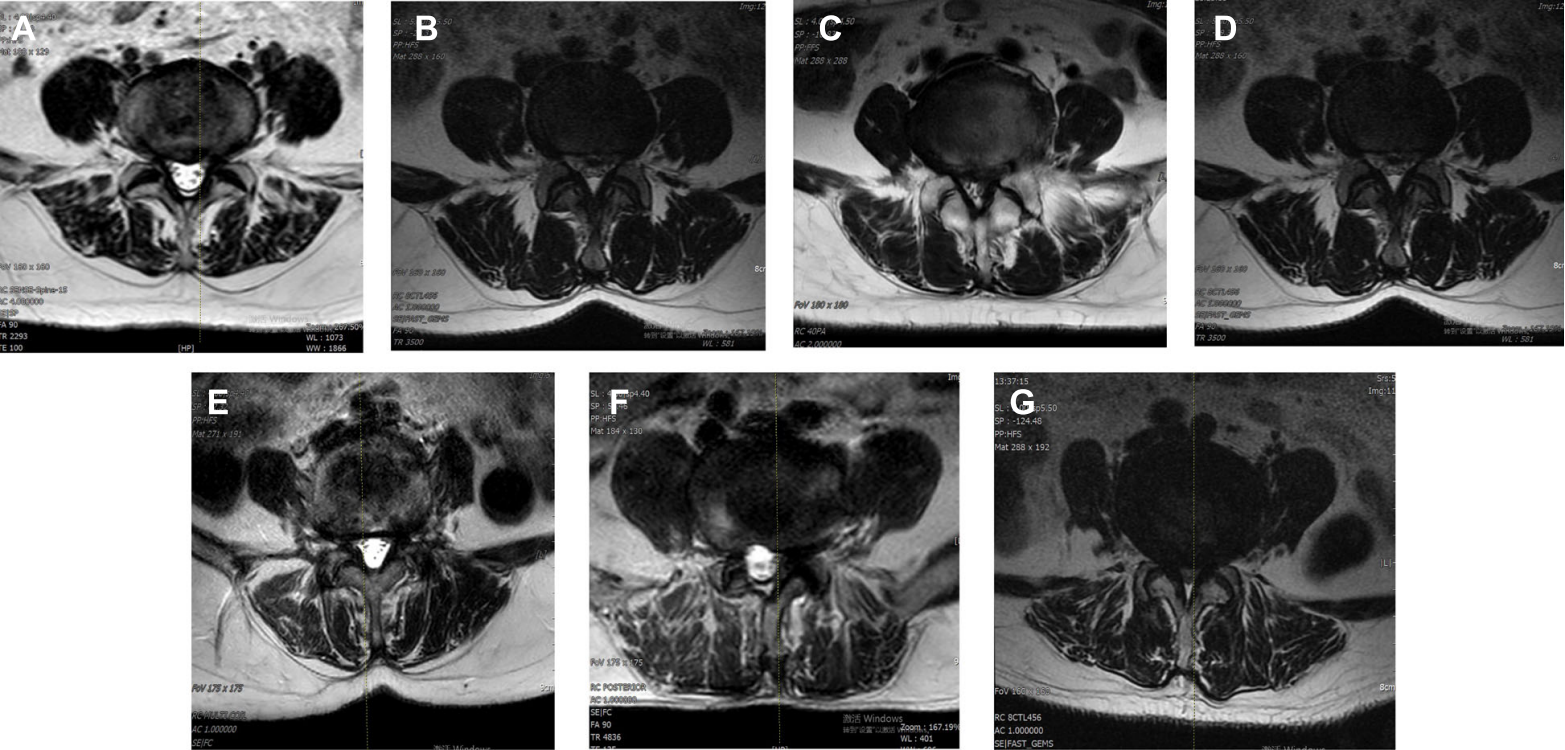
Representatives of MRI of each group of DLSS cases. **A** Control group, normal; **B** DLSS Group 6, ACD > 500 m; **C** DLSS Group 5, 400 m < ACD ≤ 500 m; **D** DLSS Group 4, 300 m < ACD ≤ 400 m; **E** DLSS Group 3, 200 m < ACD ≤ 300 m; **F** DLSS Group 5, 100 m < ACD ≤ 200 m; **G** DLSS Group 6, ACD ≤ 100 m

Absolute claudication distance (ACD), which negatively associates with the severity of spinal stenosis, was used to assess the degree of spinal stenosis since no uniform grading standard for spinal stenosis in DLSS patients has been established. The studied DLSS cases were divided into 6 groups based on ACD, with Group 1 corresponding to the shortest ACD (most severe spinal stenosis) and Group 6 corresponding to the longest ACD (least severe spinal stenosis). There are 53 cases in Group 1 (ACD ≤ 100 m), 24 cases in Group 2 (100 m < ACD ≤ 200 m), 17 cases in Group 3 (200 m < ACD ≤ 300 m), 12 cases in Group 4 (300 m < ACD ≤ 400 m), eight cases in Group 5 (400 m < ACD ≤ 500 m), and two cases in Group 6 (ACD > 500 m). MRI was used to examine the severity of spinal stenosis in each group and confirm that the severity of spinal stenosis increases from Group 1 to Group 6 (Fig. 2).

### Statistical analyses

The statistical analyses were was performed using SPSS 19.0 software (version 20.0; SPSS IBM; Armonk, NY). Normal distribution of data was performed as (`χ ± s). Spearman’s rho testing was used to analyze the correlation between the grade of L3 ~ 4, L4 ~ 5, L5 ~ S1 and the grade of spinal stenosis; the tudent’s T-test and F-test was used to compare the grade of multifidus muscle atrophy on the symptom side and the contralateral side. *P* < 0.05 was considered to show a statistically significant difference.

## Results

### Stenotic segments have more atrophic multifidus muscles than non-stenotic segments

We first examined the degree of MA in both stenotic and non-stenotic segments of the spine. DLSS cases were divided into 3 groups based on where stenosis happens: L3/4 stenosis, L4/5 stenosis, and L5/S1 stenosis. The TFCSA/TCSA ratios were calculated separately for L3/4, L4/5, and L5/S1 segments, and the mean values were compared and tested for statistical significance through T-test. We found that in each group, the TFCSA/TCSA ratios of the stenosis segments were significantly smaller than the TFCSA/TCSA ratios of the corresponding non-stenosis, indicating that MA is more severe in the stenosis segments than the non-stenosis segments (*P *< 0.05). Besides, there was no significant difference in TFCSA/TCSA ratios between non-stenosis segments (*P* > 0.05), indicating no difference in MA  (Table 2).

**Table 2:** Evaluation of TFCSA/TCSA between diseased segment and non-diseased segment

level	L3/4 stenosis (*n*=22)	L4/5 stenosis (*n*=172)	L5/S1 stenosis (*n*=28)
L3/4	74.33±2.18	76.89±2.81	77.60±2.54
L4/5	77.54±1.72^a^	75.51±2.79^a^	78.63±2.43^a^
L5/S1	77.18±1.75^bc^	77.41±2.56^bc^	75.49±2.69^bc^
*P*	*P*^a^:0.013;*P*^b^:1.249;*P*^c^:0.025	*P*^a^:0.021;*P*^b^:0.015;*P*^c^:0.9	*P*^a^:0.96;*P*^b^:0.029;*P*^c^:0.038

This table shows the mean TFCSA/TCSA ratios (%) of the L3/4, L4/5, and L5/S1 segments of each group of DLSS case. Columns correspond to groups of cases with stenosis happening in different segments. First three rows correspond to the TFCSA/TCSA ratios (%) of each segment. *P* < 0.05 was considered to show a statistically significant difference. *P, P-*value of T-test

^a^L4/5 vs L3/4, Student T-test

^b^L5/S1 vs L4/5, Student T-test

^c^L5/S1 vs L3/4, Student T-test

### The severity of MA positively correlates with the severity of spinal stenosis

**Fig. 3:**
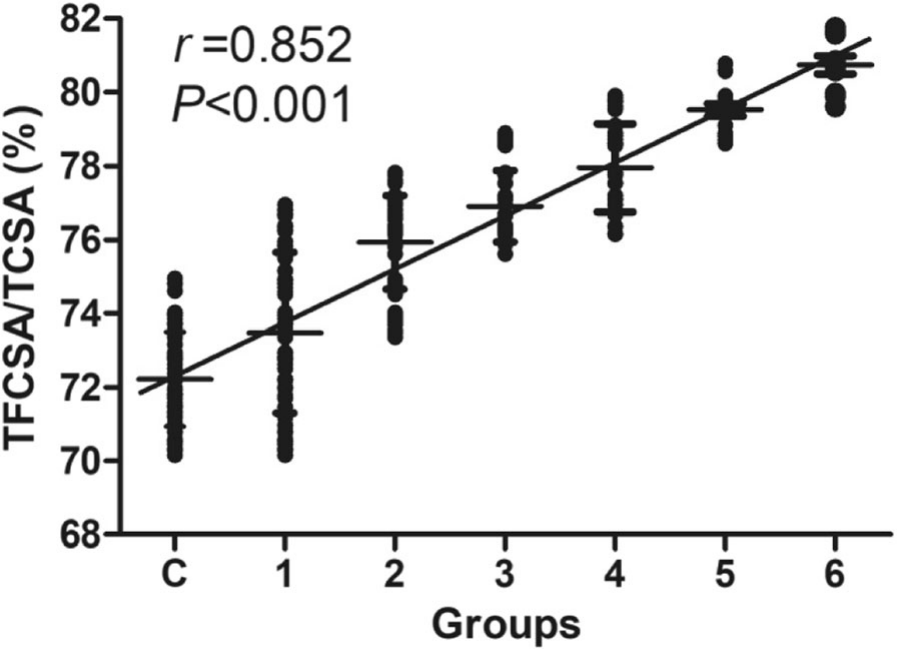
Correlation between the severity of MA and the severity of spinal stenosis. The mean TFCSA/TCSA ratios were plotted against the group number (ACD). A test for Pearson correlation was performed. *P* < 0.05 was considered to show a statistically significant difference

Next, we examined whether there is a correlation between the severity of MA and the severity of spinal stenosis. The DLSS cases were divided into 6 groups according to claudication distance (ACD) as previously mentioned. TFCSA/TCSA ratios of each group were calculated and summarized in Table 3. We performed F-test on the TFCSA/TCSA ratios and found significant differences in the TFCSA/TCSA ratios of the 6 DLSS groups (*P *< 0.05). Then we tested the Spearman’s rho testing to analyze correlation between the TFCSA/TCSA ratios and claudication distance (ACD). We found that the TFCSA/TCSA ratios positively correlated with the ACD (*P* < 0.001, *r* = 0.852) (Fig. 3), indicating that there was a strong positive correlation between the severity of MA and the severity of spinal stenosis.

**Table 3:** Evaluation of TFCSA/TCSA among the different DLSS groups

group	C	1	2	3	4	5	6
TFCSA/TCSA (%)	0.7211 ± 0.019	0.741 ± 0.017	0.769 ± 0.015	0.780 ± 0.008	0.772 ± 0.030	0.785 ± 0.002	0.785 ± 0.001
*F*		67.832					
*P*		0.001					

This table shows the mean TFCSA/TCSA ratios (%) of each of the 7 groups

*C* control group, 1-6: DLSS cases, *F* F-test results, *P* P-value of the F-test

*P* < 0.05 was considered to show a statistically significant difference

### The symptomatic sides have more atrophic multifidus muscles than the contralateral sides

**Fig. 4:**
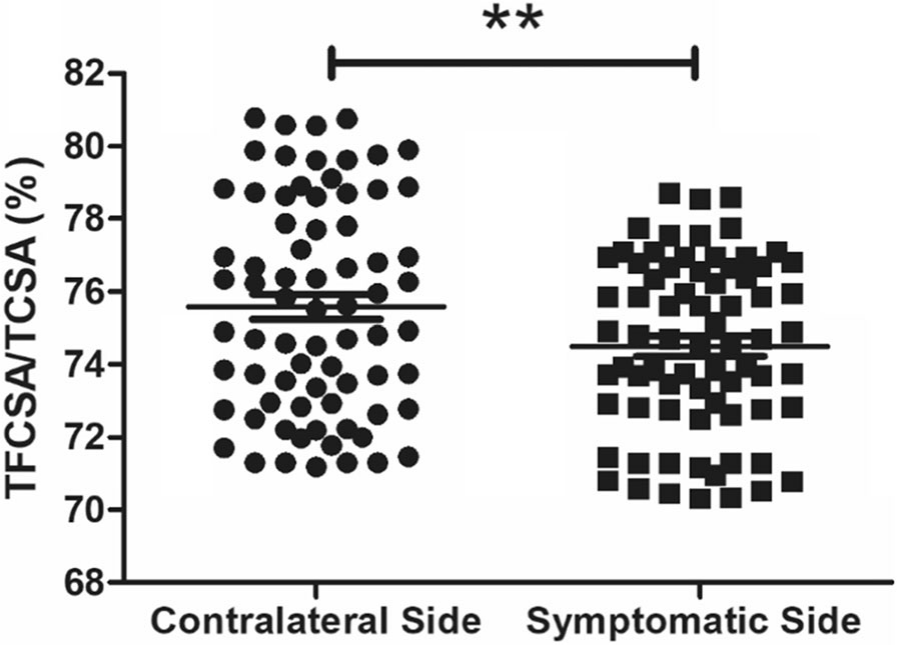
TFCSA/TCSA ratios of the symptomatic side and contralateral side. This plot shows the difference of TFCSA/TCSA ratios between the symptomatic and contralateral side of the spine; ** corresponds to *P* < 0.01

Most of the DLSS patients involved in our study have complained about asymmetric symptoms, which means the pain on one side of the spine (symptomatic side) is usually stronger than the other (contralateral side). Therefore, we examined the severity of MA on both sides by calculating the TFCSA/TCSA ratios of each side. We found that the TFCSA/TCSA ratios of the symptomatic sides are much smaller than the contralateral sides (Fig. 4). We confirmed the significance of the observed difference through the T-test (*t* = 4.128, *P* = 0.001), indicating that the symptomatic sides have more atrophic multifidus muscles than the corresponding contralateral sides.

## Discussions

In the presented study, we measured the severity of multifidus atrophy (MA) by calculating the ratios of total fat-free muscle cross-sectional area (TFCSA) to total multifidus muscle cross-sectional area (TCSA) at the L3/4, L4/5, and L5/S1 segments of the spine of 233 DLSS cases. We assessed the severity of spinal stenosis by measuring the absolute claudication distance (ACD). Through comparison, we found that the stenotic segments of the spine have more atrophic multifidus muscles compared with non-stenotic segments. The severity of MA positively correlated with the severity of spinal stenosis. Besides, we also found that the symptomatic sides of the spine have more atrophic multifidus muscles than the contralateral sides.

As one of the most common pain disorders, low back pain (LBP) has become the second most common reason for seeking medical advice [15]. LBP is defined as pain, muscle tension, or stiffness localized below the costal margin and above the inferior gluteal folds. It is usually accompanied by leg pain [17]. LBP has a high prevalence among adults, which is reported to be as high as 20.3 %, and a significant impact on life quality. LBP is often associated with lumbar disc herniation, mostly in the segments between L4/5 and L5/S1 [18, 19]. Muscle atrophy is a common pathogenic condition associated with LBP, leading to sedentary lifestyle and reduced physical activity, two common causes of chronic LBP. The atrophy of multifidus muscles, which are series of small yet powerful triangular muscle bundles located on either side of the spinal column stabilizing the lumbar spine [20], has been found to be strongly associated with LBP with an incidence of > 80 % in patients diagnosed with LBP [21]. Another common pathogenic condition causing LBP is degenerative lumbar spinal stenosis (DLSS), which is the age-related circumscribed, osteoligamentous narrowing of the spinal canal [6]. Although both muscle atrophy and DLSS have been associated with LBP, their relationship has not been examined. The presented study focused on the potential correlation between atrophy of multifidus muscles and DLSS, aiming to link the two common causes of LBP together.

Muscle atrophy is the loss of muscle mass caused by immobility, aging, malnutrition, medications, injury, or diseases [12]. Atrophy of multifidus muscles happens when the healthy muscle is replaced with fat [20]. This “fatty atrophy” can be visualized on transverse views of magnetic resonance imaging (MRI) scanning of the lumbar spine, and the ratio of the fatty area (or fat-free area) to total multifidus muscle area has been used to assess the degree of atrophy [22]. In the presented study, we used the ratio of total fat-free multifidus muscle cross-sectional area (TFCSA) to total multifidus muscle cross-sectional area (TCSA) to represent the degree of multifidus atrophy (MA). We measured the TFCSA/TCSA ratios at L3/4, L4/5, and L5/S1 segments of the spine. We found that TFCSA/TCSA ratios are always significantly smaller in the stenotic segments than non-stenotic segments, indicating more atrophic multifidus muscles are associated with spinal stenosis. One possible explanation for the severe multifidus atrophy in the stenotic segments is that the compression of the nerve root’s stenotic segment may result in the atrophy of the multifidus muscle since multifidus muscle is single-innervated by the medial branch of the posterior root of the spinal nerve [23]. Hodges et al. have found that the multifidus muscle loses its innervation, accelerates its degeneration, and is replaced by fat and connective tissue after nerve root injury, suggesting the possible linkage between the stenosis nerve root and multifidus atrophy. Another possible explanation is that the inflammatory and immune responses produced during degeneration of the intervertebral disc (happens during DLSS) will first affect the multifidus muscle [24].

Although there is still controversy about the causal relationship between multifidus atrophy and spinal stenosis, multifidus atrophy has been demonstrated to strongly correlate with spinal stenosis in the presented study. Through measuring the TFCSA/TCSA ratios of groups of cases of different degrees of spinal stenosis, we have found that severe stenosis is always accompanied by more atrophic multifidus muscles. One possible explanation for this observation may be the contraction inhibition of multifidus muscles. It has been established that spinal stenosis leads to the compression of nerve roots, a major contributing factor in the development of intermittent neurogenic claudication [25] (INC) or LBP. LBP or INC will significantly inhibit the leg or back movement, thus inhibit the contraction of the multifidus muscle. Long-term contraction inhibition will aggravate multifidus muscle atrophy and degeneration, further destabilizing the lumbar spine and falling into a feedback loop [26].

Most of the cases involved in our study have been associated with asymmetric symptoms, which means the pain on one side of the spine (symptomatic side) is usually stronger than the other (contralateral side). By measuring the TFCSA/TCSA ratios on each side, we found that the symptomatic sides are always accompanied by more atrophic multifidus muscles, which agrees with the idea of Wallwork et al., [26] that multifidus atrophy in patients with chronic low back pain is limited to the symptomatic parts, rather than systemic [14]. We think the possible reason for this observation may be that severer pain at the symptomatic side leads to the stronger contraction inhibition of multifidus muscles.

Although our results convincingly demonstrated the positive correlation between the severity of multifidus atrophy and the severity of spinal stenosis in DLSS patients, the study still has certain limitations: (1) all the cases involved in this study are Asians from the Ningxia area of China, which may not represent the global characteristics of patients with DLSS since DLSS is highly related to lifestyle; (2) multifidus muscle atrophy and spinal stenosis are both related to many factors and the influence of other factors cannot be ruled out; (3) this study only demonstrated the correlation, however, whether there is casual relationship between multifidus atrophy and spinal stenosis still remains to be determined.

## Conclusions

This study demonstrated that the severity of multifidus atrophy positively correlated with the severity of spinal stenosis in DLSS patients. Stenotic segments of the spine had more atrophic multifidus muscles and multifidus atrophy is severer at the symptomatic side of the spine.

## Data Availability

The datasets generated and/or analyzed during the current study are not publicly available but are available from the corresponding author on reasonable request.

## Abbreviations

DLSS: Degenerative Lumbar Spinal Stenosis
MA: Multifidus Atrophy
TCSA: Total multifidus muscle cross-sectional area
TFCSA: Total fat-free multifidus muscle cross-sectional area
LBP: Low back pain
ACD: Absolute claudication distance
INC: Intermittent neurogenic claudication

## Declarations

All procedures were performed in accordance with relevant guidelines’ in the manuscript.
